# Disease Progression Modeling of Estimated Glomerular Filtration Rate (eGFR): A Pharmacometrics Approach

**DOI:** 10.1111/1753-0407.70104

**Published:** 2025-06-06

**Authors:** Sohail Aziz, Sabariah Noor Harun, Siti Maisharah Sheikh Ghadzi

**Affiliations:** ^1^ School of Pharmaceutical Sciences Universiti Sains Malaysia Penang Malaysia; ^2^ Department of Clinical Pharmacy and Pharmacy Practice Faculty of Pharmacy, MAHSA University Jenjarom Malaysia

**Keywords:** disease progression, eGFR, human health, pharmacometrics, T2DM

## Abstract

**Background:**

Estimated glomerular filtration rate (eGFR) is a key clinical marker for assessing kidney complications in type 2 diabetes mellitus (T2DM). This study aimed to develop and validate a disease progression model of eGFR in Malaysian T2DM patients, with and without diabetic nephropathy (DN).

**Methods:**

Retrospective data from 251 patients (3241 observations) were analyzed using NONMEM software. Baseline eGFR was assessed without covariates, and both linear and non‐linear models were tested. Model selection was based on the likelihood ratio test (5% significance level), objective function value (OFV), visual predictive check (VPC), relative standard error, and scientific plausibility. External validation was performed using data from 109 patients.

**Results:**

A linear model best described disease progression, with a baseline eGFR of 84.6 mL/min/1.73 m^2^ and a decline rate of −0.0041 mL/min/1.73 m^2^/year. Cardiovascular disease (CVD) reduced eGFR by 1.05 mL/min/1.73 m^2^/year, while fasting blood sugar (FBS) above 7.4 mmol/L correlated with an additional decline of 0.043 mL/min/1.73 m^2^/year. Angiotensin receptor blockers (ARBs) improved eGFR by 0.4 mL/min/1.73 m^2^/year, whereas statins and metformin contributed improvements of 0.34 and 0.32 mL/min/1.73 m^2^/year, respectively. External validation confirmed model consistency with observed data.

**Conclusion:**

Glycaemic control and CVD significantly impact eGFR decline. ARBs, statins, and metformin help preserve kidney function. Effective glycaemic management is crucial in slowing kidney deterioration, especially in T2DM patients at risk for DN.

## Graphical abstract

A validated disease progression model quantified eGFR decline in T2DM patients with and without DN. Cardiovascular comorbidities and poor glycaemic control accelerated decline, while ARBs, statins, and metformin attenuated it. The model supports early, consistent use of these therapies to preserve renal function and guide individualized risk‐based interventions.

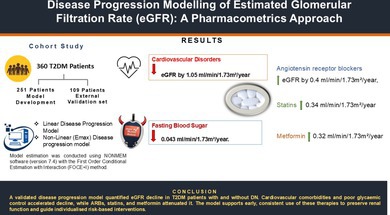

**Summary**

- The validated DP model for eGFR in T2DM patients highlighted the significant influence of glycaemic control and cardiovascular comorbidities on the progression of kidney function decline.
- The analysis highlighted the renoprotective effects of ARBs, statins, and metformin, reinforcing their role in mitigating eGFR deterioration and emphasizing the need for a targeted therapeutic approach.
- The findings emphasize the significant interaction between metabolic and cardiovascular factors in renal outcomes, highlighting the necessity of optimized glycaemic control and targeted pharmacological intervention to maintain kidney function in high‐risk T2DM populations.

## 1 Introduction

Type 2 diabetes mellitus (T2DM) is a chronic metabolic condition known to significantly increase the risk of diabetic nephropathy (DN), a progressive kidney disease characterized by the occurrence of albuminuria and a gradual decline in the estimated glomerular filtration rate (eGFR) [1]. DN remains one of the most severe complications of T2DM, with a prevalence of up to 40% in diabetic patients globally [2]. The underlying mechanisms include hyperglycaemia‐driven activation of pathways such as the formation of advanced glycation end‐products (AGEs), oxidative stress, and chronic inflammation, all of which contribute to glomerular and tubular damage [3]. However, the progression of DN can vary widely among individuals, underscoring the need for personalized approaches to treatment and management.

Recent advancements in the understanding of DN progression suggest that the disease's trajectory is highly dependent on glycaemic control, comorbid cardiovascular conditions, and pharmacological interventions [4]. Notably, patients with poor glycaemic control, as indicated by glycated hemoglobin (HbA1c) and fasting blood sugar (FBS) levels, experience accelerated decline in renal function [4]. Meanwhile, pharmacological agents such as renin‐angiotensin aldosterone system blockers (RAAS), antihyperglycaemic agents, and statins have been shown to play critical roles in slowing the decline of eGFR, thereby preserving kidney function [5, 6, 7]. RAAS medications, particularly ACE inhibitors and ARBs, mitigate eGFR reduction chiefly by alleviating glomerular hypertension via efferent arteriole dilatation. This results in a reduction of intraglomerular pressure, a crucial element in mitigating hyperfiltration‐related renal injury. Furthermore, they obstruct angiotensin II‐induced fibrotic and inflammatory pathways, thus diminishing structural damage and the advancement of fibrosis in renal tissue. A crucial mechanism is the lowering of proteinuria, a recognized indicator of disease development, which additionally safeguards against nephron loss [8, 9]. Pharmacometrics disease progression modeling offers a robust method for comprehending individual variability in eGFR decline and the temporal impacts of RAAS blockers. These models can provide personalized insights into treatment regimens by integrating patient‐specific variables, thereby aiding in enhancing long‐term patient outcomes [10].

Approximately 9% of individuals with T2DM and 19% of those over 65 years exhibit an eGFR below 45 mL/min per 1.73 m^2^. Clinical trials indicate significant progression of chronic kidney disease (CKD), with annual declines in eGFR surpassing 2.5 mL/min per 1.73 m^2^ [11]. Despite the growing body of research focused on DN, gaps remain regarding the precise predictive factors that influence the disease's progression and the impact of various pharmacological treatments over time. Moreover, while the role of albuminuria as a marker for kidney disease progression is well established, recent evidence suggests that a subset of patients may exhibit a decline in eGFR without albuminuria, complicating early diagnosis and intervention [12].

Given these complexities, disease progression (DP) modeling has emerged as a valuable tool for understanding the longitudinal changes in eGFR in T2DM patients, both with and without DN. Pharmacometrics‐based DP models, in particular, can capture the dynamic interplay between physiological biomarkers, pharmacological interventions, and disease‐modifying factors [13]. This study aims to develop a DP model for eGFR in patients with T2DM, with a focus on identifying the key covariates such as clinical biomarkers, medications, and comorbidities that influence the rate of eGFR decline. By incorporating covariates such as demographics, clinical characteristics, and medication use, the model seeks to improve the prediction of disease progression and guide more personalized treatment strategies by forecasting individual responses to medicines and pinpointing critical risk factors for complications. This method enhances drug selection, dosage, and long‐term outcome management utilizing patient‐specific information [10].

## 2 Materials and Methods

This study was a retrospective cohort analysis utilizing data from patients diagnosed with T2DM who visited diabetes clinics at hospital Sultan Abdul Halim (Kedah) and hospital Pulau Pinang (Penang), Malaysia. DN was the principal focus, and the medical records of all registered patients with T2DM, both with and without diabetic nephropathy, were examined. The data collection concentrated on the parameters of interest, specifically eGFR, for the development of the DP model.

The data was retrospectively collected from the patients' available records by analyzing the electronic database of individuals who attended the endocrinology clinic from March 2011 to December 2019. The patients were retrospectively monitored from the time of their T2DM diagnosis until the onset of DN, characterized by persistent proteinuria over three consecutive visits [14], or until the conclusion of the study. The current study had a maximum follow‐up duration of 6.6 years, uniformly distributed among all participants, with evaluations occurring at 6‐month intervals. The maximum follow‐up period of 6.6 years corresponds with the heightened likelihood of developing diabetic nephropathy over time following the diagnosis of T2DM. It has also been documented that, under normal conditions, mesangial cell expansion in the kidneys typically begins to manifest 5–9 years after the diagnosis of T2DM [2, 15]. The information extracted from the medical records included demographics, laboratory data, the onset of T2DM, and the date of diagnosis for diabetic nephropathy. Missing data was addressed using multiple imputation (fully conditional specification method) through the Statistical Package for the Social Sciences (SPSS Version 26) [16]. The inclusion criteria for subjects comprised T2DM patients, both with and without diabetic nephropathy, aged between 18 and 65 years. The exclusion criteria for the subjects included type 1 and other forms of diabetes mellitus, non‐diabetic nephropathy (renal failure without proteinuria, renal artery disease), proteinuria at the time of T2DM diagnosis, concurrent cancer, pulmonary disorders, or other chronic complications that may lead to kidney complications, as well as pregnant women.

### 2.1 Sample Size and Sampling Method

The sample size was determined after computing the average observation for each patient and the median follow‐up duration for all patients, utilizing a longitudinal data approach for linear mixed‐effects modeling (McEvoy et al. [17]). Power calculation was conducted with an 80% requirement to detect a significance level of 5%. Power calculations are utilized for the analysis of linear mixed effects models to assess the mean rate of decline [17]. In the current study, the disease progression based on HbA1c is characterized by α set at 5% and 1 − *β* at 0.8. Furthermore, *σ* was assessed as 1 fix [17], Δ was evaluated as 0.10 [18], *t*_*i*_ was established as the observation period for 10 consecutive visits every 6 months, and *t*_mean_ was calculated as the average duration for 10 observations across all patient visits, totaling 6.25 years. The calculated minimum sample size required was 148 subjects.

The current study employed a purposive sampling technique. All patients' medical records were reviewed, and those meeting the inclusion and exclusion criteria were selected as subjects for the study.

### 2.2 Pharmacometrics Disease Progression (DP) Model Building

Disease progression (DP) modeling was performed to characterize longitudinal changes in eGFR. Two structural models, a linear model and a non‐linear Emax model, were evaluated based on disease physiology assumptions. The linear model is the most basic dynamic programming model employed in this study, assuming a constant rate of change in disease status over time. It has been utilized for modeling various diseases, such as Parkinson's disease and depression [19, 20, 21]. Between‐subject variability (BSV) was modeled using an exponential structure, while residual unexplained variability (RUV) was assessed using additive, proportional, and combined error models.

Covariate analysis wasBMI‐performed sequential PPH‐on the selected base model using forward inclusion (*p* < 0.05) and backward elimination (*p* < 0.01). The final model incorporated both time‐constant and time‐varying covariates, such as demographics, blood pressure, glycated hemoglobin (HbA1c), fasting blood sugar (FBS), and pharmacological treatments.

Model estimation was conducted using NONMEM software (version 7.4) with the First Order Conditional Estimation with Interaction (FOCE+I) method [22]. Nested model comparisons were evaluated using the likelihood ratio test (LRT) at a 5% significance level (∆OFV ≥ 3.84), while non‐nested models were compared using the Akaike Information Criterion (AIC).

Model validation included internal visual predictive checks (VPC) based on 1000 simulated datasets [23] and parameter uncertainty assessment through the Sampling Importance Resampling (SIR) method, utilizing 1000 and 2000 samples as well as 500 and 1000 resamples. SIR offers the advantage of not necessitating repeated parameter estimation and being devoid of distributional assumptions [24]. Visualization and diagnostics were performed using Xpose4 and R software [25, 26].

External validation was carried out using a distinct cohort of 109 patients, applying the same study design, inclusion, and exclusion criteria. External VPCs were generated to assess predictive performance, focusing on bias and variability trends [27, 28, 29].

Additional modeling equations, detailed methodology, and statistical techniques are available in Data S1.

## 3 Results

### 3.1 Patients Demographics and Clinical Data

The current study included data from 251 patients comprising 3241 observations. The demographic data and clinical characteristics of patients are displayed in Table 1.

**TABLE 1:** Demographics and clinical characteristics of T2DM patients with and without DN (*n* = 251).

Demographics	Non‐DN (*n* = 155) *N* (%)	DN (*n* = 96) *N* (%)
Gender		
Male	93 (60.0)	62 (64.6)
Female	62 (40.0)	34 (35.4)
Ethnicity		
Malay	119 (76.8)	57 (59.4)
Chinese	7 (4.5)	13 (13.5)
Indian	29 (18.7)	26 (27.1)
Smokers	14 (9.03)	29 (30.2)
Family history		
No family history	113 (72.9)	46 (47.9)
Diabetes mellitus	40 (25.8)	43 (44.8)
Diabetes associated kidney disorders	2 (1.3)	7 (7.3)

	Non‐DN (mean ± SD)	DN (mean ± SD)
BMI, kg/m^2^	25.2 ± 2.3	26.8 ± 2.5
Age, years	49.9 ± 9.6	48.9 ± 9.7

Clinical data	Non‐DN (mean ± SD)	DN (mean ± SD)
SBP, mmHg	131 ± 8.8	137 ± 11.9
DBP, mmHg	79.1 ± 5.3	81.9 ± 8.5
HbA1c, %	9.9 ± 1.8	10.5 ± 1.8
FBS, mmol/L	8.2 ± 2.9	9.1 ± 3.1
eGFR, mL/min/1.73 m^2^	87.4 ± 16.1	82.6 ± 17.6
TG, mmol/L	2.0 ± 0.7	2.2 ± 0.9
TC, mmol/L	4.5 ± 0.8	4.8 ± 0.8

Abbreviations: BMI, body mass index; DBP, diastolic blood pressure; DN, diabetic nephropathy; eGFR, estimated glomerular filtration rate; FBS, fasting blood sugar; HbA1c, glycated Hemoglobin; SBP, systolic blood pressure; SD, standard deviation; TC, total cholesterol; TG, triglyceride.

The male gender was predominant in both the DN (64.6%) and non‐DN groups (60.0%). The Malay population constituted 119 individuals (76.8%) in the non‐DN group, whereas it represented 59.4% in the DN group, followed by the Indian demographic, as illustrated in Table 1. The mean body mass index (BMI) for the non‐DN group was 25.2 ± 2.3 kg/m^2^, whereas the DN group exhibited a marginally elevated BMI of 26.8 ± 2.5 kg/m^2^. Patients with DN exhibited a higher mean systolic blood pressure (SBP) of 137 ± 11.9 mmHg, alongside elevated levels of HbA1c (10.5% ± 1.8%) and fasting blood sugar (FBS) (9.1 ± 3.1 mmol/L) compared to non‐DN patients.

### 3.2 Development of Disease Progression Model of eGFR

#### 3.2.1 Base Model Development

The linear progression model and non‐linear disease progression models were assessed for the development of the base model for eGFR. The optimal base model was the linear disease progression model, indicated by the lowest OFV as presented in Table 2. The optimal residual model, as indicated in Table 2, was linear disease progression with additive residual unexplained variation (RUV).

**TABLE 2:** Base model development of eGFR‐based DP.

Description	Model	OFV
Linear disease progression	*S*(*t*) = *S*_0_ + α*t*	15411.0
Non‐linear disease progression	*S*(*t*) = *S*_0_ + *S*_max_ *t*/*S*_50_ + *t*	15483.0
Residual error models		
Linear DP with additive RUV	𝒀_𝒊,𝒐𝒃𝒔_ = 𝒀_𝒊,𝒑𝒓𝒆𝒅_ + 𝜺_𝒂𝒅𝒅_	15411.1
Linear DP with proportional RUV	𝒀_𝒊,𝒐𝒃𝒔_ = 𝒀_𝒊,𝒑𝒓𝒆𝒅_ × (𝟏 + 𝜺_𝒑𝒓𝒐𝒑_)	15430.0
Linear DP with combined RUV	𝒀_𝒊,𝒐𝒃𝒔_ = 𝒀_𝒊,𝒑𝒓𝒆𝒅_ × (𝟏 + 𝜺_𝒑𝒓𝒐𝒑_) + 𝜺_𝒂𝒅𝒅_	15407.5

Abbreviations: DP, disease progression; OFV, objective function value; RUV, residual unexplained variability; *S*(*t*), disease status over time; *S*_0_, baseline disease status; *S*_50_, half maximal recovery; *S*_max_, maximum recovery function; *t*, time; α, rate of change.

#### 3.2.2 Base Model

Linear disease progression was the best base model with baseline disease status of 84.5 mL/min/1.73 m^2^. The decline in eGFR was estimated as −0.0021 mL/min/1.73 m^2^/year. Additive residual error best described the residual error. Between‐subject variability (BSV) at baseline disease status was estimated as 19.3% (4%) and the rate of disease progression (BSV) was estimated at 180.8% (11%) as provided in Table 3.

**TABLE 3:** Base model parameters estimate of eGFR‐based DP.

Parameters	Typical value	RSE%
Baseline eGFR (mL/min/1.73 m^2^)	84.5	1
Rate of DP (mL/min/1.73 m^2^)	−0.0021	32
Additive residual error	6.46	3
BSV base %	19.3	9
BSV α %	180.8	20

Abbreviations: BSV, between subject variability; DP, disease progression; eGFR, estimated glomerular filtration rate; RSE, relative standard error (based on sampling‐importance resampling (SIR)); α, rate of disease progression.

#### 3.2.3 Covariate Modeling

##### 3.2.3.1 Univariate Analysis

Univariate analysis of the effects of covariates on the eGFR progression, metformin has been found to improve the eGFR by 0.038 mL/min/m^2^/year. In contrast FBS was the most significant in the decline of eGFR. In the univariate analysis, angiotensin converting enzyme inhibitors (ACEi) can be seen to improve the eGFR, while beta blockers were found to affect the eGFR negatively. Furthermore, the patients having concomitant disorders along with diabetes were also found to be significantly associated with decline in eGFR in the univariate analysis as shown in Table 4.

**TABLE 4:** Univariate analysis of eGFR‐based DP.

Parameter	∆OFV	Typical value	*p*	RSE%
Baseline	0		—	—
Metformin	−38	0.038	< 0.001	26
FBS	−26	−0.0047	< 0.001	30
Statin	−22	0.039	< 0.001	27
HbA1c	−20	−0.0079	< 0.001	37
ARBs	−20	0.0443	< 0.001	23
CVD	−14	−0.0851	< 0.001	26
ACEi	−14	0.0192	< 0.001	38
Beta blockers	−12	−0.0301	< 0.001	32
Concomitant disorders	−8	−0.0561	0.004	48
Age	−1.15	0.0006	0.28	225
Sulfonylureas	−3.97	−0.001	−0.047	46
DPP4 inhibitors	−2.15	0.014		79
Alpha glucosidase inhibitors	−16.1	0.024	< 0.001	43
PPAR agonists	−2.15	−0.019	0.14	67
Insulin	−2.85	0.028	0.09	166
Triglycerides	−1.13	−0.004	0.28	144
SBP	−1.1	0.0001	0.29	317
DBP	−5.15	0.0006	0.043	59
Hypertension	−6.15	0.018	0.04	64
CCB	−16.12	−0.023	< 0.001	40
Cholesterol	−3.45	−0.0069	0.06	72
Fibrates	−2.15	−0.025	0.14	31
Family history	−2.85	0.04	0.09	91
Social history	−3.15	0.024	0.07	93
Retinopathy	−1.15	−0.03	0.28	173

Abbreviations: ACEi, angiotensin converting enzyme inhibitors; ARBs, angiotensin receptor blockers; BMI, body mass index; CCB, calcium channel blockers; CVD, cardiovascular disorders; DBP, diastolic blood pressure; DPP4, dipeptidyl peptidase; eGFR, estimated glomerular filtration rate; FBS, fasting blood sugar; GLP, glucagon like peptide; HbA1c, glycated hemoglobin; OFV, objective function value; PPAR, peroxisome proliferators activated receptors; RSE, relative standard error (based on sampling‐importance resampling (SIR)); SBP, systolic blood pressure.

##### 3.2.3.2 Multivariate Analysis

In this stage on analysis, the significant covariates from the univariate analysis were added in forward addition manner. Covariates with highest OFV difference were retained for the next part of the analysis while those with OFV difference of less than 3.84 for one df were excluded. Five covariates from the forward addition were retained. In the backward elimination, no covariates were excluded as all the covariates fulfill the criteria of increase in OFV by more than 6.63 (*p* > 0.01) as shown in Table 5.

**TABLE 5:** Multivariate analysis of eGFR‐based DP.

N	Parameters	Model	∆OFV	*p*
Forward addition				
1	Base	*S*(*t*) = *S*_0_ + *αt*	0	—
2	Metformin	*S*(*t*) = *S*_0_ *(1 + *αt*) × (1 + θ_1_*metformin)	−38	< 0.001
3	FBS	*S*(*t*) = *S*_0_*(1 + *αt*) × (1 + θ_1_* metformin + θ_2_*(FBS‐7.4))	−20	< 0.001
4	ARBs	*S*(*t*) = *S*_0_*(1 + *αt*) × (1 + θ_1_* metformin + θ_2_*(FBS‐7.4) + θ_3_*ARBS)	−17	< 0.001
5	Statin	*S*(*t*) = *S*_0_*(1 + *αt*) × (1 + θ_1_* metformin + θ_2_*(FBS‐7.4) + θ_3_*ARBS + θ_4_*statin)	−16	< 0.001
6	CVD	*S*(*t*) = *S*_0_*(1 + *αt*) × (1 + θ_1_* metformin + θ_2_*(FBS‐7.4) + θ_3_*ARBS + θ_4_*statin + θ_5_*CVD)	−14	< 0.001
Backward elimination				
7	Metformin	*S*(*t*) = S_0_*(1 + *αt*) × (1 + θ_1_*(FBS‐7.4) + θ_2_*ARBS + θ_3_*statin + θ_4_*CVD)	32.7	< 0.001
8	FBS	*S*(*t*) = *S*_0_*(1 + *αt*) × (1 + θ_1_* metformin + θ_2_*ARBS + θ_3_*statin + θ_4_*CVD)	20.87	< 0.001
9	ARBs	*S*(*t*) = *S*_0_*(1 + *αt*) × (1 + θ_1_* metformin + θ_2_*(FBS‐7.4) + θ_3_*statin + θ_4_*CVD)	13.17	< 0.001
10	Statin	*S*(*t*) = *S*_0_*(1 + *αt*) × (1 + θ_1_* metformin + θ_2_*(FBS‐7.4) + θ_3_*ARBS + θ_4_*CVD)	16.28	< 0.001
11	CVD	*S*(*t*) = *S*_0_*(1 + *αt*) × (1 + θ_1_* metformin + θ_2_*(FBS‐7.4) + θ_3_*ARBS + θ_4_*statin)	14.37	< 0.001

Abbreviations: ∆OFV, difference in objective function value; ARBs, angiotensin receptor blockers; CVD, cardiovascular disorders; FBS, fasting blood sugar; *S*(*t*), eGFR at time *t*; *S*_0_, eGFR at baseline; *t*, time since diagnosis; α, slope parameter indicating rate of eGFR changes over time; θ, estimated effects of covariate on eGFR.

#### 3.2.4 Final eGFR DP Model

The effects of the final model parameters on the disease progression is presented in Table 6. Baseline status of eGFR was 84.6 mL/min/1.73 m^2^, while the typical value of rate of disease progression was estimated as −0.0041 mL/min/1.73 m^2^/year. CVD concurrent to diabetes results in decrease of eGFR by 1.05 mL/min/1.73 m^2^/year, while every one unit rise in FBS above 7.4 mmol/L was associated with decrease in eGFR by 0.043 mL/min/1.73 m^2^/year. Angiotensin receptor blockers ARBs have shown to improve the eGFR by 0.4 mL/min/1.73 m^2^/year, while statin and metformin improved the eGFR by 0.34 and 0.32 mL/min/1.73 m^2^/year, respectively as presented in Table 6. Overall, the model showed good results of RSE below 35%. RSE is a measure of the uncertainty in the estimated effect of each covariate. The estimated RSE from model findings indicates relatively precise estimate, indicating a robust effect of the covariates on the HbA1c levels.

**TABLE 6:** Final model parameters estimate.

Parameters	Typical value	RSE%
Baseline eGFR (mL/min/1.73 m^2^)	84.6	2
Rate of DP (mL/min/1.73 m^2^)	−0.0041	20
Metformin	0.029	28
FBS	−0.0038	34
ARBs	0.0361	32
Statin	0.0314	33
CVD	−0.0925	25
Proportional residual error	6.29	3
BSV base %	18.7	9
BSV α %	157.8	16

Abbreviations: ARBs, angiotensin receptor blockers; BSV, between subject variability; CVD, cardiovascular disorders; DP, disease progression; eGFR, estimated glomerular filtration rate; FBS, fasting blood sugar; RSE, relative standard error (based on sampling importance resampling (SIR)); α, rate of disease progression.

### 3.3 Model Validation

#### 3.3.1 Internal Validation

**FIGURE 1:**
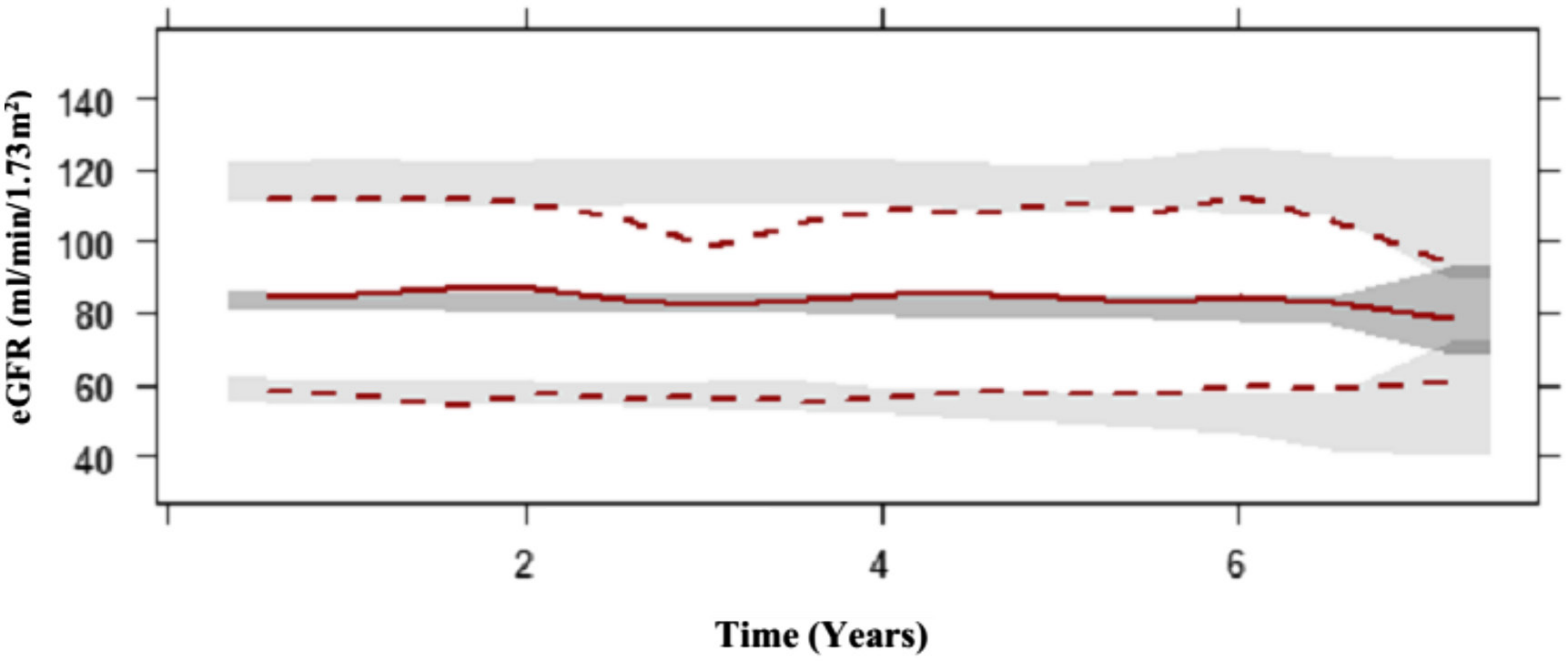
Visual predictive check for eGFR‐based disease progression model, The *x*‐axis represents years since diagnosis of T2DM, Top, middle and bottom red dashed and solid lines represent 95th, 50th and 5th percentiles of the observed data with the gray shaded areas presenting the 95% confidence intervals for the 95th, 50th and 5th percentiles of the 1000 simulated datasets.

**FIGURE 2:**
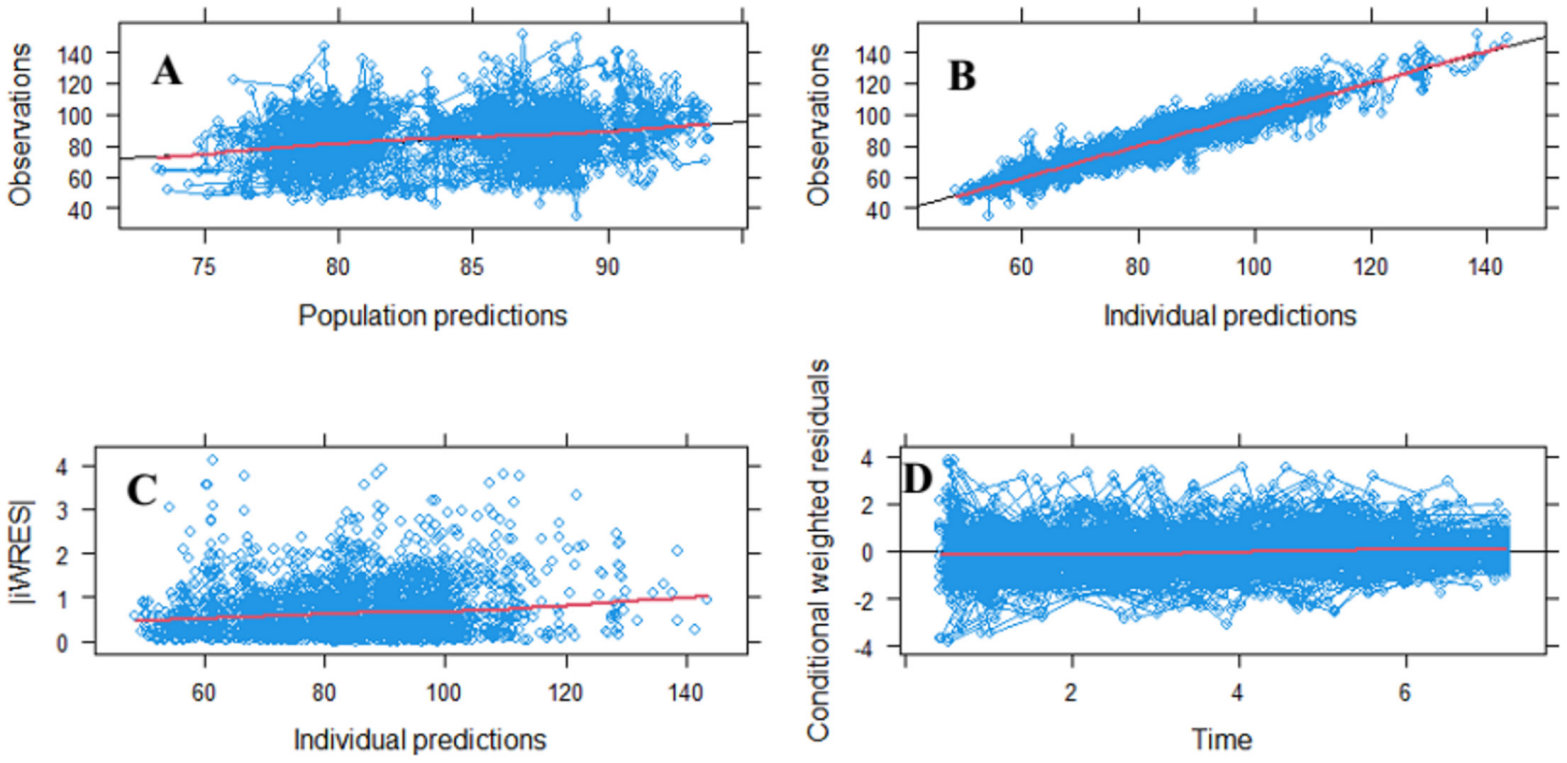
Goodness‐of‐fit plots for the final linear progression model, observed eGFR (ml/min/1.73 m^2^) predicted versus population‐predicted eGFR (A), observed eGFR predicted versus individual‐predicted eGFR (B), Individual weighted residuals (IWRES) vs. individual prediction (C), Conditional weighted residuals versus time (years) (D).

Visual predictive checks and goodness‐of‐fit plots are illustrated in Figures 1 and 2 respectively. The median observed data overlaid the simulated data, with the exception of underprediction at 1.5–2 years post T2DM diagnosis. The upper percentile overpredicted the data at 3 years post T2DM diagnosis. These variances in predictions can be associated with the variability in eGFR in every single patient on a different pharmacological treatment strategy as well as the variability in eGFR during those visits to the clinic.

The goodness of fit plots (GOF) for the final model indicate good agreement between the individual predicted eGFR and the population predicted eGFR (Plot A). As provided in Plot B, the model performance in capturing the underlying relationship on the basis of eGFR in individual predictions vs. observations is satisfactory and indicates good performance of the model. Plot C, indicating individual weighted residuals (IWRES) vs. individual predictions, has the red line consistently lying above 0, which indicates that the model may overestimate relatively smaller values and may underestimate relatively larger values in the case of eGFR, but does not undermine the model's capacity to accurately reflect general trends and variability within clinically acceptable parameters. Plot D, indicating the residual plot vs. individual predictions, shows that most of the residual error is distributed in the same manner around the reference line, which indicates that the model prediction accurately captured the observed pattern of eGFR at different time points, as shown in Figure 2.

#### 3.3.2 External Validation

**FIGURE 3:**
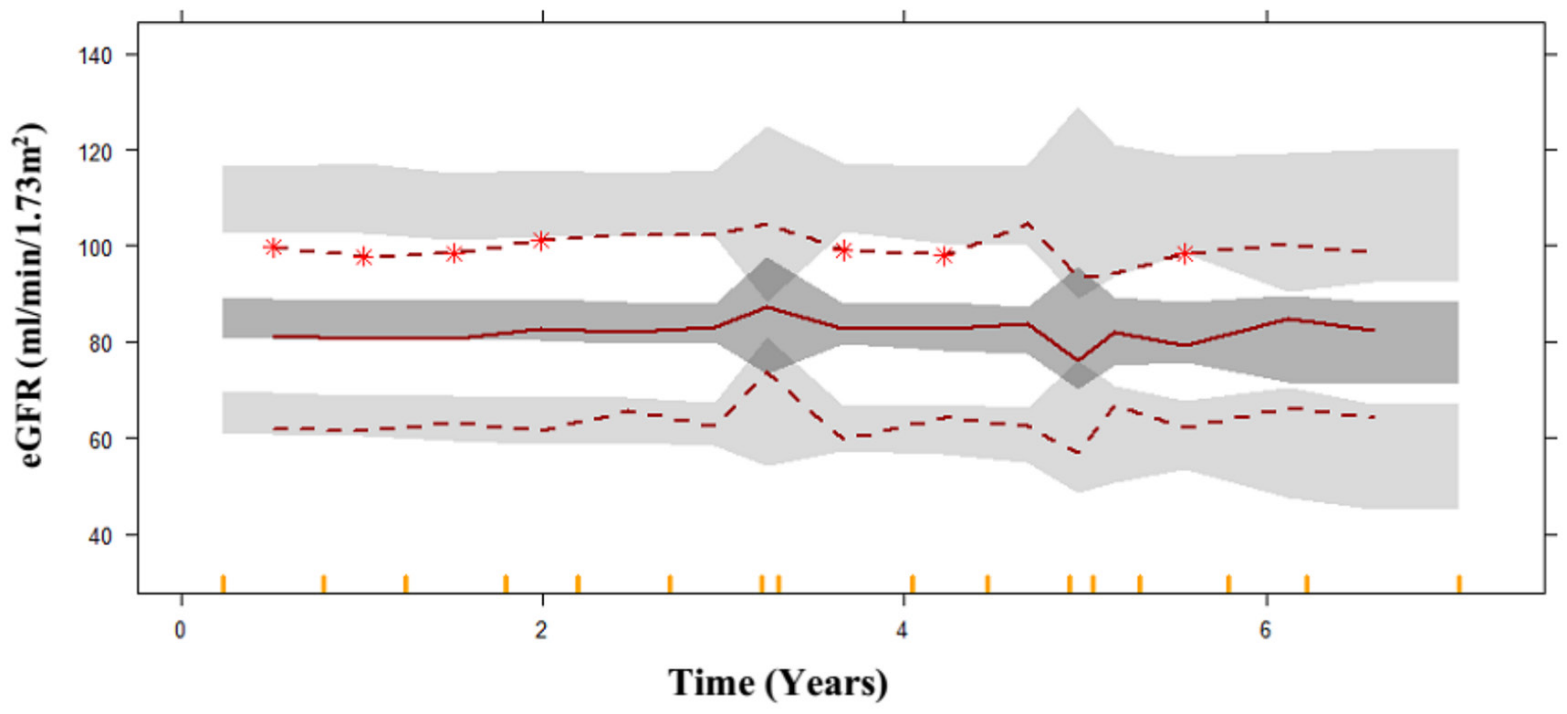
External validation visual predictive check for eGFR‐based disease progression model, the *x*‐axis represents years since diagnosis of T2DM, Top, middle, and bottom red dashed and solid lines represent 95th, 50th and 5th percentiles of the observed data with the gray shaded areas presenting the 95% confidence intervals for the 95th, 50th and 5th percentiles of the 1000 simulated datasets. This figure allows assessment of the model's predictive performance in an independent cohort by comparing observed percentiles to model‐predicted intervals.

The final model estimates were externally validated on a dataset comprising 109 patients. VPC was generated on the basis of the external dataset with 1000 simulations. The median and lower percentile well overlaid the prediction interval, indicating the model's predictions are generally consistent with the observed data within that range. The upper percentile was overpredicted, in that case model tends to provide higher predictions than what is observed in that range of values. In this case the model is overly optimistic or assumes higher values than actually occur in the external dataset as shown in Figure 3. This may indicate the model's propensity to extrapolate above the reported data range, a prevalent difficulty when utilizing models on new datasets. Nonetheless, the model continues to offer significant insights into the overarching trend of disease progression and can be enhanced to increase precision in the upper range.

## 4 Discussion

Existing literature indicates that a significant percentage of diabetics exhibit renal dysfunction, as seen by abnormal eGFR, even in the absence of albuminuria, with recent studies suggesting an increasing prevalence in type 2 diabetes [30]. Hence, the present study aimed at developing a disease progression model based on eGFR. Linear disease progression best described the progression of eGFR over time. Baseline eGFR was 84.6 mL/min/1.73 m^2^. Among the drugs, metformin, ARBs, and statins were significantly associated with the improvement in eGFR, while FBS and cardiovascular disorders were significantly associated with a decline in eGFR over time.

The present study observed an association between an increase in FBS levels above 7.4 mmol/L and a subsequent decrease in eGFR by 0.043 mL/min/1.73 m^2^/year. In a 2017 Cochrane study by Ruospo et al., it was observed that FBS less than 6.7 mmol/L in intense versus less stringent FBS management was not related to significant changes in the risk of blood creatinine doubling [31]. In contrast to the aforementioned findings, a study conducted on a sample of 183 049 Korean adults diagnosed with diabetes and using antihyperglycemic drugs revealed that the hazard ratios associated with the occurrence of new‐onset albuminuria and major cardiovascular events were found to be at their lowest when FBS levels were between 6.1 and 7.8 mmol/L. Most patients had the lowest hazard ratios for all‐cause mortality at FBS levels between 6.1 mmol/L and less than 7.8 mmol/L [32]. These results are in accordance with the findings of the present study indicating the significant role of FBS on eGFR trend over time. As previous findings of intensive glucose management have shown better effects on kidney function and decrease in all‐cause mortality, still the range of FBS that can be targeted in the general population is controversial and may need to be individualized [33, 34]. In a similar study conducted on Chinese diabetic patients, it was observed that FBS 6.1 to 7.0 mmol/L was associated with 1.83 times the risk of kidney function disorder, while greater than 7 mmol/L was associated with a risk of 2.51 times of kidney function decline [35]. The mechanism associated with FBS on kidney function was evaluated in a study by Ikee et al., where they demonstrated that FBS was significantly associated with interstitial fibrosis [36].

The primary goal of treating individuals with T2DM is to control hyperglycaemia and prevent microvascular and macrovascular complications. Metformin remains the first‐line pharmacological agent, acting mainly through reducing hepatic glucose production and enhancing peripheral glucose uptake [37]. Emerging evidence suggests a reno‐protective effect of metformin, attributed to its ability to mitigate oxidative stress in podocytes by inhibiting NADPH oxidase and enhancing free‐radical defense systems [38]. In this study, metformin use was associated with an improvement in eGFR by 0.32 mL/min/1.73 m^2^/year, irrespective of the administered dosage [39].

Recent revisions in clinical guidelines have broadened metformin's use to patients with mild and, cautiously, moderate renal impairment (eGFR 45–60 mL/min/1.73 m^2^ and even < 45 mL/min/1.73 m^2^ in selected cases) [40]. Mechanistic studies suggest that metformin may diminish renal fibrosis and improve kidney function via the AMPK signaling pathway [41], although appropriate dosing remains critical [42]. Metformin's reno‐protective effects are believed to stem from anti‐inflammatory, antioxidant, and anti‐fibrotic mechanisms, independent of glycaemic control [43].

In this study, ARBs were associated with an improvement in eGFR by 0.4 mL/min/1.73 m^2^/year. The renin‐angiotensin system (RAS) plays a pivotal role in the pathophysiology of diabetic nephropathy, and clinical trials such as IDNT and RENAAL have established the benefits of ARBs in slowing renal disease progression independent of blood pressure control [15, 44]. ARBs have also shown efficacy in reducing proteinuria and mitigating the risk of overt nephropathy [45]. Trials like IRMA‐2, IDNT, RENAAL, and ORIENT further support the use of ARBs, particularly in hypertensive CKD patients with proteinuria [46].

Guidelines recommend ARBs or ACEis as first‐line therapy for CKD management [45]. A meta‐analysis indicated that ARB monotherapy could prevent 11 cases of ESRD and regress albuminuria in 118 individuals per 1000 treated, though risks such as acute kidney injury and hyperkalaemia remain considerations [6]. While ARBs show significant reno‐protective benefits, the choice between monotherapy and dual RAS blockade continues to be debated based on efficacy and safety profiles.

The present study observed a decline in eGFR of 1.05 mL/min/1.73 m^2^/year. In a study on a large cohort of patients based on the effects of CVD on kidney disorders observed similar findings were observed. The study observed a decline in kidney function (Serum creatinine increase by 0.4 mg/dL) in 3.8% of patients, while 2.3% of patients developed kidney disorders during 9 years of follow‐up. Furthermore, the decline in eGFR was independently associated with CVD (HR: 1.13–1.45) [47]. Similarly, in accordance with the present study results, a study of 4380 US participantsobserved that CVD was independently associated with a decline in eGFR (OR: 1.55), hence suggesting the role of underlying renal atherosclerosis responsible for the decline in eGFR [48]. It has been observed and well established in the literature that CVD and kidney disorders are interrelated, and the disorder of one organ causes the dysfunction of the other organ. The traditional risk factors for the development of a decline in kidney functions are those that lead to CVD, i.e., hypertension, dyslipidaemia, and diabetes [49, 50]. As observed in the present study, statins were associated with an improvement in yearly eGFR decline by 0.34 mL/min/1.73 m^2^. Our results are in line with Esmeijer et al., which stated that the usage of statins decreased the rate of yearly kidney function decline by 0.61 mL/min/1.73 m^2^ and decreased proteinuria by 0.58 mg/g. Similarly, Su et al. demonstrated that statins were associated with a 0.41 mL/min/1.73 m^2^ slower yearly eGFR decline and a reduction of 0.65 mg/g in proteinuria [51, 52]. Although the exact mechanism of statin in renoprotection is unknown but in addition to reducing serum cholesterol, statins may also protect the kidney, and the increase in endothelial NO production may be one of the probable pathways [53]. The study by Kimura et al., in contrast to the present study findings, found no evidence of improvement in eGFR by the use of atorvastatin in patients at risk of developing kidney dysfunction, which can be associated with the patients' background as the patients were older age having eGFR lower than 60 mL/min/1.73 m^2^ [53]. Furthermore, in another study on the effects of statin on eGFR decline, it was observed that long term treatment for 1–3 years with statins was significantly associated with improvement in eGFR by 0.50 mL/min/1.73 m^2^ [54]. Similarly, another study evaluating the efficacy of statins observed that high potency statin treatment can delay the decline in kidney function and may also reduce CVD [55]. Furthermore, a post hoc analysis of 6 double blind RCT observed that the effects of statin (atorvastatin) on eGFR is dose dependent but at the same time improved kidney function and lowered CV risks [56]. In contrast, a population‐based cohort study of 128 140 statin users, assessed statin use at interval of 30 days and demonstrated that aggressive management with statin is associated with acute kidney injury in older male patients although the magnitude of risk is small [57]. Based on the present study findings and available literature, it is advisable to monitor patients' lipid profiles and start statin treatment in diabetic patients above the age of 40 years, regardless of their cholesterol level, to avoid the complications of developing CVD and kidney dysfunction.

The novelty of the present study lies in the development and external validation of a pharmacometrics disease progression model for eGFR decline using real‐world longitudinal data from Malaysian T2DM patients. Unlike traditional epidemiological analyses, our model quantitatively characterizes the dynamic effects of pharmacological treatments and clinical covariates, including the identification of biomarkers associated with eGFR deterioration. Furthermore, the external validation using an independent patient cohort strengthens the model's utility for personalized therapeutic decision‐making in DN.

## 5 Limitation

A significant restriction pertains to the accessibility and quality of the data utilized in the investigation. The conclusions of the present study rely heavily on the retrospective data acquired from endocrine clinics, making them dependent on the correctness, completeness, and reliability of the available records. The restricted number of participants in the study may affect the generalizability of the findings to a broader community of T2DM patients. Observational studies are susceptible to confounding variables and biases, such as selection and information bias, that may affect the observed correlations. The missing data were imputed using multiple imputation that may have introduced bias. However, the missing data were minimal and primarily affected time‐varying laboratory variables such as FBS, HbA1c, SBP, BMI, triglycerides, and cholesterol measurements. The proportion of missingness was less than 5% for each variable. Given the low extent of missing data and the use of multiple imputation by the fully conditional specification method, we anticipate minimal bias introduced into the longitudinal modeling.

## 6 Conclusions

Present study developed and validated DP model of eGFR in T2DM patients with and without DN. The findings observed the impact of glycaemic control and cardiovascular disorders on eGFR decline, while highlighting the role of treatments with ARBs, statins, and metformin in preserving eGFR. The study is suggestive of the importance of optimal glycaemic control with an appropriate treatment strategy in minimizing kidney function decline, specifically in T2DM patients at risk of developing DN. The clinical implications of this study suggest that consistent use of metformin, ACEi/ARBs, and statins can significantly attenuate the decline in kidney function in patients with T2DM. The pharmacometrics modeling approach provides a quantitative framework to predict individual risk trajectories and supports proactive therapeutic strategies. Future studies should aim to further explore the mechanisms underlying these treatment effects and validate the DP model across broader populations to enhance its clinical applicability.

## Ethics Statement

Ethical approval was secured from the Medical Research and Ethics Committee (MREC) under the reference number (NMRR‐18‐3134‐43 563). The study protocols received approval from the Human Research Ethics Committee (JEPeM) at Universiti Sains Malaysia, identified by the number USM/JEPeM/18100614. The acquired data are maintained in confidentiality and restricted solely to the investigators. Patient's names and any other identities are not identifiable; all data are stored anonymously. The data reports were assigned numerical codes to ensure anonymity; the collected reports are securely stored and are not accessible to individuals not directly involved in this study.

## Conflicts of Interest

The authors declare no conflicts of interest.

## Supporting information


**Data S1.** Supporting Information.

## References

[1] M. K. Sagoo and L. Gnudi , Diabetic Nephropathy: An Overview, vol. 2 (Springer, 2020), 45–56. 10.1007/978-1-4939-9841-8_1 31701441

[2] R. Z. Alicic , M. T. Rooney , and K. R. Tuttle , “Diabetic Kidney Disease: Challenges, Progress, and Possibilities,” Clinical Journal of the American Society of Nephrology 12, no. 12 (2017): 2032–2045. 28522654 10.2215/CJN.11491116 PMC5718284

[3] K. Umanath and J. B. Lewis , “Update on Diabetic Nephropathy: Core Curriculum 2018,” American Journal of Kidney Diseases 71, no. 6 (2018): 884–895. 29398179 10.1053/j.ajkd.2017.10.026

[4] N. Wang and C. Zhang , “Recent Advances in the Management of Diabetic Kidney Disease: Slowing Progression,” International Journal of Molecular Sciences 25, no. 6 (2024): 3086. 38542060 10.3390/ijms25063086 PMC10970506

[5] A. M. Patti , R. V. Giglio , N. Papanas , M. Rizzo , and A. A. Rizvi , “Future Perspectives of the Pharmacological Management of Diabetic Dyslipidemia,” Expert Review of Clinical Pharmacology 12, no. 2 (2019): 129–143. 30644763 10.1080/17512433.2019.1567328

[6] S. C. Palmer , D. Mavridis , E. Navarese , et al., “Comparative Efficacy and Safety of Blood Pressure‐Lowering Agents in Adults With Diabetes and Kidney Disease: A Network Meta‐Analysis,” Lancet 385, no. 9982 (2015): 2047–2056. 26009228 10.1016/S0140-6736(14)62459-4

[7] S. Aziz , S. M. S. Ghadzi , S. A. S. Sulaiman , N. H. M. Hanafiah , and S. N. Harun , “Can Newer Anti‐Diabetic Therapies Delay the Development of Diabetic Nephropathy?,” Journal of Pharmacy & Bioallied Sciences 13, no. 4 (2021): 341–351. 35399797 10.4103/jpbs.jpbs_497_21 PMC8985833

[8] M. Nandave , “Introduction of Renin‐Angiotensin‐Aldosterone System (RAAS),” in Angiotensin‐Converting Enzyme Inhibitors vs. Angiotensin Receptor Blockers: A Critical Analysis of Antihypertensive Strategies: A Machine‐Generated Literature Overview (Springer Nature Singapore, 2024), 1–72.

[9] S. Alshahrani , “Renin–Angiotensin–Aldosterone Pathway Modulators in Chronic Kidney Disease: A Comparative Review,” Frontiers in Pharmacology 14 (2023): 1101068. 36860293 10.3389/fphar.2023.1101068 PMC9970101

[10] S. Aziz , S. N. Harun , S. A. S. Sulaiman , and S. M. S. Ghadzi , “Pharmacometrics Approaches and Its Applications in Diabetes: An Overview,” Journal of Pharmacy & Bioallied Sciences 13, no. 4 (2021): 335–340. 35399800 10.4103/jpbs.jpbs_399_21 PMC8985840

[11] K. G. Garlo , W. B. White , G. L. Bakris , et al., “Kidney Biomarkers and Decline in eGFR in Patients With Type 2 Diabetes,” Clinical Journal of the American Society of Nephrology 13, no. 3 (2018): 398–405. 29339356 10.2215/CJN.05280517 PMC5967667

[12] M. Oshima , M. Shimizu , M. Yamanouchi , et al., “Trajectories of Kidney Function in Diabetes: A Clinicopathological Update,” Nature Reviews Nephrology 17, no. 11 (2021): 740–750. 34363037 10.1038/s41581-021-00462-y

[13] S. F. Cook and R. R. Bies , “Disease Progression Modeling: Key Concepts and Recent Developments,” Current Pharmacology Reports 2 (2016): 221–230. 28936389 10.1007/s40495-016-0066-x PMC5602534

[14] D. Etana Tola , Z. B. Bayissa , T. A. Desissa , L. K. Solbana , A. H. Tesfaye , and B. F. Eba , “Determinants of Diabetic Nephropathy Among Adult Diabetic Patients on Follow‐Up at Public Hospitals in Addis Ababa, Ethiopia: A Case‐Control Study,” SAGE Open Medicine 12 (2024): 20503121231218890. 38222310 10.1177/20503121231218890 PMC10787527

[15] N. M. Selby , B. Bmbs , M. Dm , M. W. Taal , and B. Mmed , “An Updated Overview of Diabetic Nephropathy: Diagnosis, Prognosis, Treatment Goals and Latest Guidelines,” Diabetes, Obesity and Metabolism 22, no. S1 (2020): 3–15. 10.1111/dom.14007 32267079

[16] F. E. Tan , S. Jolani , and H. Verbeek , “Guidelines for Multiple Imputations in Repeated Measurements With Time‐Dependent Covariates: A Case Study,” Journal of Clinical Epidemiology 102 (2018): 107–114. 29964148 10.1016/j.jclinepi.2018.06.006

[17] L. K. McEvoy , S. D. Edland , D. Holland , D. J. Hagler, Jr. , J. C. Roddey , and C. Fennema‐Notestine , “Neuroimaging Enrichment Strategy for Secondary Prevention Trials in Alzheimer Disease,” Alzheimer Disease & Associated Disorders 24, no. 3 (2010): 269–277. 20683184 10.1097/WAD.0b013e3181d1b814 PMC2929320

[18] J. Nojima , S. Meguro , N. Ohkawa , M. Furukoshi , T. Kawai , and H. Itoh , “One‐Year eGFR Decline Rate Is a Good Predictor of Prognosis of Renal Failure in Patients With Type 2 Diabetes,” Proceedings of the Japan Academy. Series B, Physical and Biological Sciences 93, no. 9 (2017): 746–754. 29129852 10.2183/pjab.93.046 PMC5743850

[19] M. A. Felmlee , M. E. Morris , and D. E. Mager , “Mechanism‐Based Pharmacodynamic Modeling,” Computational Toxicology 1 (2012): 583–600. 10.1007/978-1-62703-050-2_21 PMC3684160 23007443

[20] E. Y. Shang , M. A. Gibbs , A. E. Jaren , et al., “Evaluation of Structural Models to Describe the Effect of Placebo Upon the Time Course of Major Depressive Disorder,” Journal of Pharmacokinetics and Pharmacodynamics 36, no. 1 (2009): 63–80. 19205853 10.1007/s10928-009-9110-3

[21] T. C. Vu , J. G. Nutt , and N. H. G. Holford , “Progression of Motor and Nonmotor Features of Parkinson's Disease and Their Response to Treatment,” British Journal of Clinical Pharmacology 74, no. 2 (2012): 267–283. 22283961 10.1111/j.1365-2125.2012.04192.x PMC3630747

[22] S. Beal , L. Boeckmann , R. Bauer , and L. Sheiner , NONMEM User's Guides (ICON PLC, 2009), 1989–2009.

[23] M. Bergstrand , A. C. Hooker , J. E. Wallin , and M. O. Karlsson , “Prediction‐Corrected Visual Predictive Checks for Diagnosing Nonlinear Mixed‐Effects Models,” AAPS Journal 13, no. 2 (2011): 143–151. 21302010 10.1208/s12248-011-9255-z PMC3085712

[24] A. G. Dosne , M. Bergstrand , K. Harling , and M. O. Karlsson , “Improving the Estimation of Parameter Uncertainty Distributions in Nonlinear Mixed Effects Models Using Sampling Importance Resampling,” Journal of Pharmacokinetics and Pharmacodynamics 43, no. 6 (2016): 583–596. 27730482 10.1007/s10928-016-9487-8 PMC5110709

[25] R. J. Keizer , M. O. Karlsson , and A. Hooker , “Modeling and Simulation Workbench for NONMEM: Tutorial on Pirana, PsN, and Xpose,” CPT: Pharmacometrics & Systems Pharmacology 2, no. 6 (2013): 1–9. 10.1038/psp.2013.24 PMC3697037 23836189

[26] M. Fidler , J. J. Wilkins , R. Hooijmaijers , et al., “Nonlinear Mixed‐Effects Model Development and Simulation Using Nlmixr and Related R Open‐Source Packages,” CPT: Pharmacometrics & Systems Pharmacology 8, no. 9 (2019): 621–633. 31207186 10.1002/psp4.12445 PMC6765694

[27] S. E. Bleeker , H. A. Moll , E. W. Steyerberg , et al., “External Validation Is Necessary in Prediction Research: A Clinical Example,” Journal of Clinical Epidemiology 56, no. 9 (2003): 826–832. 14505766 10.1016/s0895-4356(03)00207-5

[28] W. Zhao , F. Kaguelidou , V. Biran , et al., “External Evaluation of Population Pharmacokinetic Models of Vancomycin in Neonates: The Transferability of Published Models to Different Clinical Settings,” British Journal of Clinical Pharmacology 75, no. 4 (2013): 1068–1080. 23148919 10.1111/j.1365-2125.2012.04406.x PMC3612725

[29] M. R. Muda , O. Albitar , S. N. Harun , S. A. S. Sulaiman , I. A. H. Ali , and S. M. S. Ghadzi , “A Time‐To‐Event Modelling of Sputum Conversion Within Two Months After Antituberculosis Initiation Among Drug‐Susceptible Smear Positive Pulmonary Tuberculosis Patients: Implementation of Internal and External Validation,” Tuberculosis 148 (2024): 102553. 39094294 10.1016/j.tube.2024.102553

[30] O. Buyadaa , D. J. Magliano , A. Salim , D. N. Koye , and J. E. Shaw , “Risk of Rapid Kidney Function Decline, all‐Cause Mortality, and Major Cardiovascular Events in Nonalbuminuric Chronic Kidney Disease in Type 2 Diabetes,” Diabetes Care 43, no. 1 (2020): 122–129. 31796570 10.2337/dc19-1438 PMC7411281

[31] M. Ruospo , V. M. Saglimbene , S. C. Palmer , et al., “Glucose Targets for Preventing Diabetic Kidney Disease and Its Progression,” Cochrane Database of Systematic Reviews 2017, no. 6 (2017): CD010137. 10.1002/14651858.CD010137.pub2 PMC6481869 28594069

[32] H. H. Jung , “Evaluation of Serum Glucose and Kidney Disease Progression Among Patients With Diabetes,” JAMA Network Open 4, no. 9 (2021): e2127387. 34586368 10.1001/jamanetworkopen.2021.27387 PMC8482057

[33] V. Perkovic , H. L. Heerspink , J. Chalmers , et al., “Intensive Glucose Control Improves Kidney Outcomes in Patients With Type 2 Diabetes,” Kidney International 83, no. 3 (2013): 517–523. 23302714 10.1038/ki.2012.401

[34] M. G. Wong , V. Perkovic , J. Chalmers , et al., “Long‐Term Benefits of Intensive Glucose Control for Preventing End‐Stage Kidney Disease: ADVANCE‐ON,” Diabetes Care 39, no. 5 (2016): 694–700. 27006512 10.2337/dc15-2322

[35] X. Wang , F. Fan , J. Jia , et al., “Association of Different Glucose Traits With Kidney Function Decline Risk in a Chinese Community‐Based Population Without Chronic Kidney Disease,” Therapeutics and Clinical Risk Management 14 (2018): 1725–1734. 30271157 10.2147/TCRM.S167233 PMC6147541

[36] R. Ikee , Y. Hamasaki , M. Oka , et al., “Glucose Metabolism, Insulin Resistance, and Renal Pathology in Non‐Diabetic Chronic Kidney Disease,” Nephron. Clinical Practice 108, no. 2 (2008): c163–c168. 18259103 10.1159/000115329

[37] C. C. R. Betônico , S. M. O. Titan , M. L. C. Correa‐Giannella , M. Nery , and M. Queiroz , “Management of Diabetes Mellitus in Individuals With Chronic Kidney Disease: Therapeutic Perspectives and Hic Control,” Clinics 71, no. 1 (2016): 47–53. 26872083 10.6061/clinics/2016(01)08 PMC4732385

[38] A. Piwkowska , D. Rogacka , M. Jankowski , M. H. Dominiczak , J. K. Stepiński , and S. Angielski , “Metformin Induces Suppression of NAD(P)H Oxidase Activity in Podocytes,” Biochemical and Biophysical Research Communications 393, no. 2 (2010): 268–273. 20123087 10.1016/j.bbrc.2010.01.119

[39] D. Kawanami , Y. Takashi , and M. Tanabe , “Significance of Metformin Use in Diabetic Kidney Disease,” International Journal of Molecular Sciences 21, no. 12 (2020): 4239. 32545901 10.3390/ijms21124239 PMC7352798

[40] S. Bell , B. Farran , S. McGurnaghan , et al., “Risk of Acute Kidney Injury and Survival in Patients Treated With Metformin: An Observational Cohort Study,” BMC Nephrology 18, no. 1 (2017): 163. 28526011 10.1186/s12882-017-0579-5 PMC5437411

[41] Z. Lv and Y. Guo , “Metformin and Its Benefits for Various Diseases,” Frontiers in Endocrinology 11 (2020): 191. 32425881 10.3389/fendo.2020.00191 PMC7212476

[42] E. Neven , B. Vervaet , K. Brand , et al., “Metformin Prevents the Development of Severe Chronic Kidney Disease and Its Associated Mineral and Bone Disorder,” Kidney International 94, no. 1 (2018): 102–113. 29716795 10.1016/j.kint.2018.01.027

[43] C. S. Boddepalli , S. D. Gutlapalli , V. K. Lavu , et al., “The Effectiveness and Safety of Metformin Compared to Sulfonylureas in Diabetic Nephropathy: A Systematic Review,” Cureus 14, no. 12 (2022): e32286. 36628027 10.7759/cureus.32286 PMC9822529

[44] S. B. Gurley and T. M. Coffman , “The Renin‐Angiotensin System and Diabetic Nephropathy,” in Seminars in Nephrology, vol. 27 (WB Saunders, 2007), 144–152. 17418683 10.1016/j.semnephrol.2007.01.009

[45] M. Burnier , S. Lin , L. Ruilope , G. Bader , S. Durg , and P. Brunel , “Effect of Angiotensin Receptor Blockers on Blood Pressure and Renal Function in Patients With Concomitant Hypertension and Chronic Kidney Disease: A Systematic Review and Meta‐Analysis,” Blood Pressure 28, no. 6 (2019): 358–374. 31392910 10.1080/08037051.2019.1644155

[46] E. Imai , J. C. N. Chan , S. Ito , et al., “Effects of Olmesartan on Renal and Cardiovascular Outcomes in Type 2 Diabetes With Overt Nephropathy: A Multicentre, Randomised, Placebo‐Controlled Study,” Diabetologia 54, no. 12 (2011): 2978–2986. 21993710 10.1007/s00125-011-2325-z PMC3210358

[47] E. F. Elsayed , H. Tighiouart , J. Griffith , et al., “Cardiovascular Disease and Subsequent Kidney Disease,” Archives of Internal Medicine 167, no. 11 (2007): 1130–1136. 17563020 10.1001/archinte.167.11.1130

[48] M. G. Shlipak , R. Katz , B. Kestenbaum , L. F. Fried , D. Siscovick , and M. J. Sarnak , “Clinical and Subclinical Cardiovascular Disease and Kidney Function Decline in the Elderly,” Atherosclerosis 204, no. 1 (2009): 298–303. 18848325 10.1016/j.atherosclerosis.2008.08.016 PMC2696894

[49] L. P. Gregg and S. S. Hedayati , “Management of Traditional Cardiovascular Risk Factors in CKD: What Are the Data?,” American Journal of Kidney Diseases 72, no. 5 (2018): 728–744. 29478869 10.1053/j.ajkd.2017.12.007 PMC6107444

[50] P. Krisanapan , P. Pattharanitima , C. Thongprayoon , and W. Cheungpasitporn , “Recent Advances in Understanding of Cardiovascular Diseases in Patients With Chronic Kidney Disease,” Journal of Clinical Medicine 11, no. 16 (2022): 4653. 36012887 10.3390/jcm11164653 PMC9409994

[51] K. Esmeijer , O. M. Dekkers , J. W. de Fijter , F. W. Dekker , and E. K. Hoogeveen , “Effect of Different Types of Statins on Kidney Function Decline and Proteinuria: A Network Meta‐Analysis,” Scientific Reports 9, no. 1 (2019): 16632. 31719617 10.1038/s41598-019-53064-x PMC6851118

[52] X. Su , L. Zhang , J. Lv , et al., “Effect of Statins on Kidney Disease Outcomes: A Systematic Review and Meta‐Analysis,” American Journal of Kidney Diseases 67, no. 6 (2016): 881–892. 26905361 10.1053/j.ajkd.2016.01.016

[53] G. Kimura , M. Kasahara , K. Ueshima , et al., “Effects of Atorvastatin on Renal Function in Patients With Dyslipidemia and Chronic Kidney Disease: Assessment of Clinical Usefulness in CKD Patients With Atorvastatin (ASUCA) Trial,” Clinical and Experimental Nephrology 21, no. 3 (2017): 417–424. 27392909 10.1007/s10157-016-1304-6 PMC5486454

[54] D. Nikolic , M. Banach , S. Nikfar , et al., “A Meta‐Analysis of the Role of Statins on Renal Outcomes in Patients With Chronic Kidney Disease. Is the Duration of Therapy Important?,” International Journal of Cardiology 168, no. 6 (2013): 5437–5447. 24016544 10.1016/j.ijcard.2013.08.060

[55] V. G. Athyros , N. Katsiki , A. Karagiannis , and D. P. Mikhailidis , “Statins Can Improve Proteinuria and Glomerular Filtration Rate Loss in Chronic Kidney Disease Patients, Further Reducing Cardiovascular Risk. Fact or Fiction?,” Expert Opinion on Pharmacotherapy 16, no. 10 (2015): 1449–1461. 26037614 10.1517/14656566.2015.1053464

[56] L. Vogt , S. Bangalore , R. Fayyad , et al., “Atorvastatin Has a Dose‐Dependent Beneficial Effect on Kidney Function and Associated Cardiovascular Outcomes: Post Hoc Analysis of 6 Double‐Blind Randomized Controlled Trials,” Journal of the American Heart Association 8, no. 9 (2019): e010827. 31020900 10.1161/JAHA.118.010827 PMC6512126

[57] M. Tonelli , A. M. Lloyd , A. K. Bello , et al., “Statin Use and the Risk of Acute Kidney Injury in Older Adults,” BMC Nephrology 20, no. 1 (2019): 1–9. 30909872 10.1186/s12882-019-1280-7 PMC6434639

