# Supplementary material for: Disease Progression Modeling of Estimated Glomerular Filtration Rate (eGFR): A Pharmacometrics Approach
Source: J Diabetes. 2025 Jun 6;17(6):e70104. doi: 10.1111/1753-0407.70104 (PMC12141788; doi:10.1111/1753-0407.70104)
Supplement: Supplementary file 1 — Data S1. Supporting Information. [file JDB-17-e70104-s001.docx]

**Supplementary Material 1 – Modelling Methods and Equations**

**Base Model Development and Equations**

Two DP models were evaluated: the linear model and the Emax model.

Linear model equation:

|  | **S(𝑡) = S_0_ + αt** | Equation 1 |
| --- | --- | --- |

The exponential error structure depicted in equation 2 is the most frequently employed function for introducing subject variability into a model and has been used in the present study.

|  | 𝑷_𝒊_ = 𝑻𝑽𝑷_i_ × 𝒆^𝜼^_pi_ | Equation 2 |
| --- | --- | --- |
|  |  |  |

The residual error represents the variance between the expected and observed values. The residual unexplained variability (RUV) was calculated using additive, proportional or combined error models.

The following equations were used for the additive, proportional, and combined residual error respectively;

|  | | 𝒀_𝒊, 𝒐𝒃𝒔_ = 𝒀_𝒊, 𝒑𝒓𝒆𝒅_ + 𝜺_𝒂𝒅𝒅_ | Equation 3 | |
| --- | --- | --- | --- | --- |
|  | 𝒀_𝒊, 𝒐𝒃𝒔_ = 𝒀_𝒊, 𝒑𝒓𝒆𝒅_ × (𝟏 + 𝜺_𝒑𝒓𝒐𝒑_) | | | Equation 4 |

|  | 𝒀_𝒊, 𝒐𝒃𝒔_ = 𝒀_𝒊, 𝒑𝒓𝒆𝒅_ × (𝟏 + 𝜺_𝒑𝒓𝒐𝒑_) + 𝜺_𝒂𝒅𝒅_ | Equation 5 |
| --- | --- | --- |

In the Emax model, the patient status at any time “t” is described as the sum of the baseline status S_0_ and some recovery function that has a maximum of S_max_. The time to half maximal recovery is S_50_ as shown in equation 6.

S(𝑡) = S_0_ + S_max_t/S_50_+t Equation 6

**Covariates Modelling**

|  | S(𝑡) = S_0_ + αt × (1+θ_1_cov_1_+ θ_2_cov_2._+ θ_n_cov_n_) | Equation 7 |
| --- | --- | --- |

In DP modelling, potential time-varying and time-constant explanatory covariates that could affect or predict disease trajectory were evaluated by incorporating each explanatory factor into the model for disease progression. The covariate modelling phase was conducted on the optimal base model selected from the base model development process.

S(𝑡) represents the disease status, S_0_ denotes the baseline disease status (or baseline clinical marker), α signifies the slope parameter, and t indicates the time elapsed since the initial observation of the disease. θ represents the parameter for covariates, whereas θ_n_ denotes the nth covariate.

**Model estimation and selection**

Parameters were estimated utilising NONMEM software [22] via the First Order Conditional Estimation with Interaction (FOCE+I) for DP (ADVAN6 TOL=6) to derive maximum likelihood estimates of the parameters. The foundational model for DP modelling delineates the natural progression of DP and serves as a framework for integrating supplementary factors or covariates that may affect DP, including demographic attributes (e.g., age, gender, marital status, ethnicity, and social history) or clinical metrics (e.g., blood pressure, glycated haemoglobin (HbA1c), fasting blood sugar (FBS), triglycerides, cholesterol). The likelihood ratio test (LRT) was employed to differentiate between nested models, utilising a significance level (α) of 5%. According to LRT, the objective function value (OFV) is presumed to follow a chi-square distribution, and a reduction of 3.84 in OFV between hierarchical models with one degree of freedom (df) (such as the inclusion of a covariate is deemed statistically significant at α=5%). Model selection was determined through numerical diagnostics utilising the objective function value (OFV), goodness-of-fit (GOF) plots, precision of parameter estimates, and scientific validity. If the OFV among models with comparable degrees of freedom is not significantly different, the model deemed more biologically plausible was chosen. The Akaike Information Criterion (AIC) is employed to select the optimal model in the context of a non-nested model.

**Model validation and evaluation**

To determine that the model adequately represents the data or not, the final models underwent validation testing. Visual predictive checks (VPC) were conducted during model development to assess the predictive performance of the models [23]. To internally validate the final model, VPC was conducted using 1000 simulated datasets.

Furthermore, model performance was assessed via relative standard error (RSE) derived from the sample importance resampling (SIR) technique, utilising 1000 and 2000 samples as well as 500 and 1000 resamples. SIR offers the advantage of not necessitating repeated parameter estimation and being devoid of distributional assumptions. SIR is a viable approach for assessing parameter uncertainty, particularly in scenarios where alternative methods are ineffective, including situations involving limited datasets, meta-analysis, or highly nonlinear models [24]. The data results were visualised using the Xpose4 package (version 4.6.1, Uppsala, Sweden) within R software (version 4.1.1) [25,26].

**External validation**

One method to assess the reliability of model parameters is through external validation of predictive models, which involves evaluating the model on a dataset from an independent study [27]. In the current study, data collection was conducted separately for the subjects engaged in model development and external validation. The data from patients utilised for model validation was excluded from the model development to guarantee that it originates from a distinct population. The dataset utilised for model development comprised 251 subjects, while a distinct cohort of 109 subjects was incorporated for external validation. The study design, sampling method, and inclusion and exclusion criteria mirror those of the primary study, with a follow-up duration of 7 years. During the external validation of the DP model, diagnostic plots were produced. VPCs were created to evaluate model performance by comparing observed data from an external dataset with simulated data from the model. The VPC plots were assessed for systematic bias, including overprediction or underprediction, by comparing observed data with anticipated intervals. The plots were evaluated to ascertain if the model well represented the key trends and variability patterns of an independent external dataset, assuring adequate coverage of observed data within the predicted percentiles [28,29]
